# Alexithymic Trait, Painful Heat Stimulation, and Everyday Pain Experience

**DOI:** 10.3389/fpsyt.2015.00139

**Published:** 2015-10-07

**Authors:** Olga Pollatos, Anja Dietel, Harald Gündel, Stefan Duschek

**Affiliations:** ^1^Health Psychology, Institute of Psychology, University of Ulm, Ulm, Germany; ^2^Department for Endocrinology, Diabetes and Vascular Medicine, Academic Teaching Hospital Munich Bogenhausen, Munich, Germany; ^3^Clinic for Psychotherapy and Psychosomatics, University Clinic of Ulm, Ulm, Germany; ^4^Institute of Applied Psychology, UMIT – University for Health Sciences, Medical Informatics and Technology, Hall in Tirol, Austria

**Keywords:** heat pain, pain perception, alexithymia, self-reported pain, depression, somatic symptoms

## Abstract

**Background:**

Alexithymia was found to be associated with a variety of somatic complaints, including somatoform pain symptoms. This study addressed the question of whether the different facets of alexithymia are related to responses in heat pain stimulation and its interrelations with levels of everyday pain as assessed by self-report.

**Methods:**

In the study, sensitivity to heat pain was assessed in 50 healthy female participants. Alexithymia facets were assessed by the Toronto Alexithymia Scale. Pain threshold and tolerance were determined using a testing the limits procedure. Participants, furthermore, rated subjective intensities and unpleasantness of tonic heat stimuli (45.5–47.5°C) on visual analog scales and on a questionnaire. Possible confounding with temperature sensitivity and mood was controlled. Everyday pain was assessed by self-report addressing everyday pain frequency, intensity, and impairment experienced over the last 2 months.

**Results:**

Main results were that the facets of alexithymia were differentially associated with pain perception. The affective scale “difficulties in describing feelings” was associated with hyposensitivity to pain as indicated by higher pain tolerance scores. Furthermore, everyday pain frequency was related to increased alexithymia values on the affective scale “difficulties in identifying feelings,” whereas higher values on the cognitive alexithymia scale “externally oriented thinking” were related to lower pain impairment and intensity.

**Conclusion:**

We conclude that the different facets of alexithymia are related to alternations in pain processing. Further research on clinical samples is necessary to elucidate whether different aspects of alexithymia act as a vulnerability factor for the development of pain symptoms.

## Introduction

Alexithymia is characterized by a marked difficulty to identify, describe, and express one’s emotions (1, 2) and has been related to a broad range of somatic and psychiatric disorders {e.g., alcoholism, drug addiction, somatoform disorders [see Ref. (3, 4)]}. Self-report measures like the Toronto Alexithymia Scale (TAS) (5), the most widely used and well-validated assessment tool (5–7), assess alexithymia with three main facets, namely difficulties in identifying feelings (DIF), difficulties in describing feelings (DDF), and externally oriented thinking or a preoccupation with the details of external events (EOT).

Alexithymia hampers effective regulation of emotion and interacts with the perception of emotional stimuli (8, 9) and it has been suggested (10) that these deficits may, in turn, result in a negative affect state that fosters a hypervigilance toward somatic sensations and increased report of somatic complaints. Different studies could demonstrate that healthy participants scoring high on alexithymia report more somatic complaints (11), leading to the assumption that alexithymia is associated with over-reporting of physical symptoms, including pain.

Previous studies support this view: a meta-analytic review of 18 studies (12) found significant positive correlations between alexithymia (facet DIF) and measures of somatic symptoms. Katz and co-authors (13) hypothesized that the association between alexithymia and measures of self-reported pain might above all reflect difficulties in emotion regulation. Also, Porcelli and colleagues (14) reported that DIF subscale was correlated with quality descriptors of pain in patients. Recent data (15, 16) support this notion, as, e.g., in the general population, higher levels of alexithymia are associated with higher risk of having chronic pain.

When investigating other measures than self-report results are not consistent. Nyklicek and Vingerhoets (11) used painful electric stimulation and found alexithymics to be more sensitive to experimentally induced pain, similar to data from Huber and co-authors (17), while other studies did not support this interpretation (17). On the contrary, Moriguchi and colleagues (18) could demonstrate that high alexithymic participants scored lower on pain intensity when watching others in pain.

Importantly, all previous studies lack to assess both self-report measures of everyday pain in terms of somatic complains and perceived impairment and objective pain sensitivity to disentangle subjective experience, perceptual, and affective steps of pain processing. The focus of this study was the subclinical range of alexithymia as target variable, assessed in a healthy population. We hypothesized that the emotional subcomponents of alexithymia, especially the DIF scale, would be positively associated with everyday pain. We, therefore, assumed positive correlations of the different measures of everyday pain and the emotional facets of alexithymia. Additionally, we aimed at investigating the interaction of alexithymia subfacets with experimentally induced heat pain. Having in mind that various studies demonstrated emotional disturbances in alexithymia (19, 20), we hypothesized that the emotionally modulated pain tolerance scores interact with alexithymia, especially the emotional facets. Based on empirical data, it still remains unclear in which direction the assumed correlations between alexithymia subscales on experimentally induced pain are to be expected and how such differential effects of alexithymia subscales are interrelated to everyday pain.

## Materials and Methods

### Ethic statement

Experiments were conducted in accordance with the Declaration of Helsinki with the approval of the local ethics committees (ethic committee of the Department of Psychology at the University of Munich). In accordance with the local ethic committee, all participants provided their written informed consent. They received €20 for their participation.

### Participants

Healthy volunteers were recruited from introductory psychology courses and screened for suitability prior to the main experiment. Of those who contacted the department only about 10% were excluded due to the following exclusion criteria. Criteria for exclusion comprised relevant physical diseases, acute, or chronic pain of any kind (i.e., any pain associated with either drug intake or physical therapy and therefore not allowing a categorization as healthy participant), psychiatric disorders as well as the use of any drugs (except of contraceptives). As only 10 males volunteered, the final sample was restricted to females only. Health status was assessed by anamnestic interview and a comprehensive medical questionnaire. Fifty female participants took part in the main experiment. All selected subjects were confirmed right-handed by means of the Edinburgh Handedness Inventory (21). No participant terminated the experiment early.

The experimental procedure was the following: after the screening (variable time prior to the main experiment), all participants arrived at the laboratory. All experiments took place between 10 a.m. and 4 p.m. First, all participants filled in the questionnaires. Then, the main experiment with the experimental pain examination took place.

### Questionnaires

All participants filled in the following questionnaires: Alexithymia was assessed using the TAS (2). The TAS-20 is the most psychometrically valid and commonly used self-report measurement of alexithymia (5, 22) consisting of 20 items rated on a 5-point scale with total scores ranging from 20 to 100. The different facets assessed are DIF, DDF, and externally oriented thinking or a preoccupation with the details of external events (EOT). There is growing empirical evidence that these facets probably refer to different correlates (23–26) with high intercorrelations between the DIF and DDF subscales and lower intercorrelations to the EOT subscale (7, 25, 26).

Current emotional state was examined using the Zerssen Mood Scale [“Befindlichkeits-Skala” (27)] which is a 28-item self-rating scale (range 0–56) widely used in German speaking countries with good reliability and validity. It was used in former studies using a similar experimental procedure [e.g., Ref. (28)], leading to a high variance of participants’ answers. It is, therefore, an ideal tool to control for mood interaction on, e.g., pain perception, as described both for negative as well as positive mood in the literature [see, e.g., Ref. (29, 30)].

### Pain induction and quantification

Thermal stimulation was performed using a Thermal Sensory Analyzer (TSA II, Medoc Advanced Medical Systems, Israel). A contact thermode (surface 30 mm × 30 mm) was attached to the volar surface of the left forearm. The thermode was digitally controlled employing the software WinTSA and CoVAS (Medoc, Israel). First, warm and cold sensory thresholds were determined. For this purpose, the temperature of the thermode rose or fell at a rate of 1°C/s, beginning at 32°C. Participants were instructed to press a response key as soon as they noted the first sensation of warm or cold. Temperature returned to 32°C immediately after the keystroke at a rate of 10°C/s. Five warm followed by five cold stimuli were applied with inter-stimulus intervals randomly ranging from 4 to 7 s.

Pain sensitivity and tolerance were quantified using a testing the limits procedure. Once again, the temperature increased at 1°C/s from a baseline of 32°C. To determine pain threshold, participants pressed the key when the sensation “started to become painful.” In case of pain tolerance, the key was pressed when subjects could “no longer tolerate the pain.” Five trials were performed for each condition (inter-stimulus interval 10 s, return rate 10°C/s) [c.f., Ref. (31)]. In order to obtain estimates for thermal thresholds, pain sensitivity and pain tolerance, the temperatures at which the keystrokes took place were averaged across the five trials of each condition.

Ratings on subjective pain intensity were obtained using tonic heat stimulation. Therefore, five stimuli of temperatures ranging from 45.5 to 47.5°C were presented (pseudorandom sequence: 45.5, 46.5, 47, 46, and 47.5°C). Stimulus duration was 60 s with each stimulus being preceded by a 60 s baseline of 32°C (increasing and return rate 7°C/s). Stimulus temperatures were chosen according to previous studies [e.g., Ref. (28, 31, 32)], suggesting that this temperature range is suitable to detect interindividual differences in subjective heat pain in healthy samples, thereby avoiding bottom or ceiling effects. In order to prevent sensitization, the thermode was repositioned between the trials. The participants’ subjective pain experience was quantified using two 10-cm line visual analog scales (VASs) referring to the sensory and affective aspects of pain (“How strong/unpleasant was the pain?” range 0–10). The anchor points of the scales were marked “not at all” and “extremely.” The scales were presented immediately after each stimulus. In order to avoid redundancy and to enhance the reliability of the measurement, the ratings for sensory and affective pain were averaged across the five stimuli. After the last stimulus, the overall pain experience during tonic heat stimulation was rated on the Pain Sensation Scale [“Schmerzempfindungsskala” (33)]. This is a well-established self-rating questionnaire comprising a list of 24 adjectives related either to the sensory (e.g., pungent, biting) or affective (e.g., horrible, unsupportable) dimension of pain (33).

### Everyday pain experience

Participants were asked to rate everyday pain frequency, intensity and impairment experienced over the last 2 months examining frequent main categories. The aim was to assess everyday pain in a broad range. For this purpose, participants first were asked whether or not they had experienced headache, back pain, stomach ache, limb pain, joint pain, menstrual pain, teeth ache, and other pain during the last 2 months. Then, for each pain category reported they rated the frequency using the following categories: less than once a month, 1–3 times a month, once a week, twice a week, 2–5 times a week, daily, and several times a day. In addition, they were asked to mark the experienced pain intensity for each pain type on a 10-cm VAS ranging from “no pain” to “highest imaginable pain.” A further 10-cm VAS was used to evaluate the impairment in daily life caused by pain ranging from “not at all” to “extremely.” Impairment in daily life was defined as disturbance in professional life, in free time or in social contacts.

Pain frequency categories were ranked as follows: less than once a month: rank 1, 1–3 times a month: rank 2, once a week: rank 3, twice a week: rank 4, 2–5 times a week: rank 5, daily: rank 6, and several times a day: rank 7. Then, for each checked pain type (e.g., headache) ranks were added to obtain a sum score of pain frequency. We calculated a mean frequency score of everyday pain across all pain categories as well as a mean everyday pain intensity score by summarizing all single scores.

### Data analysis

Main focus of all analyses is the detailed association between alexithymia facets, pain measures, and everyday pain. Kolmogorov–Smirnov tests implemented in SPSS version 21 were used to test for normal distribution of all outcome variables. In case that no significant deviations from normal distribution were found, partial correlation analyses were calculated between pain threshold, pain tolerance, measures of everyday pain and the three subscales of the TAS. Possible confounding with warm thresholds, age, body mass index (BMI), and mood was controlled for. In the second step, four hierarchical regression analyses were carried out in which consecutively pain threshold, pain tolerance and everyday pain frequency and intensity served as criterion. In accordance to former methodological approaches [see, e.g., Ref. (11)], warm threshold, age, and mood as well as the three subscales of the TAS were used in all regression analyses as predictors.

## Results

### Sample characteristics

Sample characteristics as well as pain indices are depicted in Table 1. Participants were aged 27.8 ± 4.7 years (mean ± SD). They had a mean TAS total score of 39.7 (±9.1; DIF mean 13.2; DDF mean 10.8; EOT mean 15.7). Mean score in the Zerssen Mood Scale was 13.5 ± 8.2. Kolmogorov–Smirnov tests showed no significant deviation from normal distribution for all TAS subscales as well as the mean mood scale (DIF: test score 0.12, *p* = 0.07; DDF: test score 0.11, *p* = 0.20; EOT: test score 0.12, *p* = 0.08; mean mood scale: test score 0.12, *p* = 0.07).

**Table 1:** **Sample characteristics (*N* = 50 females)**.

	Mean	SD
Age (years)	27.8	4.7
BMI (kg/m^2^)	21.7	3.2
**Questionnaires**
Toronto Alexithymia Scale		
Total score (range 20–100)	39.7	9.1
DIF (range 7–35)	13.2	4.1
DDF (range 5–25)	10.8	3.9
EOT (range 8–40)	15.7	4.3
Zerssen Mood Scale (range 0–56)	13.5	8.2
**Pain threshold and pain tolerance**
Warm sensitivity (°C)	35.0	1.7
Cold sensitivity (°C)	31.2	0.3
Experimental mean pain threshold to heat stimuli (°C)	44.3	2.6
Experimental mean pain tolerance to heat stimuli (°C)	48.3	1.1
**Evaluation of tonic heat stimuli**
Perceived pain intensity (range 0–10)	6.2	1.7
Perceived pain unpleasantness (range 0–10)	6.4	1.6
Affective scale (SES, range 14–56)	27.5	7.2
Sensory scale (SES, range 10–40)	24.1	4.9
**Everyday pain**
Sum of pain frequency (range 0–56)	5.7	3.1
Mean pain intensity (range 0–10)	1.2	0.9
Impairment (range 0–10)	2.6	1.9

### Experimental pain assessment

The mean values of the subjects’ warm and cold thresholds were 34.9 ± 1.7 and 31.1 ± 0.3°C, respectively (see Table 1). As initial stimulus temperature was 32°C, subjects were able to detect warming by 2.9°C and cooling by 0.9°C on average. Pain threshold to heat stimulation was 44.3 ± 2.6°C, mean pain tolerance was 48.3 ± 1.1°C.

The aggregated VAS ratings of subjective pain experience to standardized heat stimuli are also summarized in Table 1. The mean perceived pain intensity was 6.0 ± 1.7, the mean perceived unpleasantness was 6.3 ± 1.6. The overall pain experience during the whole tonic heat stimulation (rated on the Pain Sensation Scale) was assessed concerning sensory and affective dimensions. The mean scores are summarized in Table 1. Pain threshold, pain tolerance, and the VAS ratings were tested for normal distribution; they did not differ significantly from normal distribution (Kolmogorov–Smirnov tests: pain threshold: test score 0.11, *p* = 0.10; pain tolerance: test score 0.12, *p* = 0.06; pain intensity: test score 0.11, *p* = 0.17; pain unpleasantness: test score 0.11, *p* = 0.16).

### Everyday pain experience

Nearly all participants (in average 92%) reported to have experienced at least one type of pain during the last 2 months, referring to several pain types, such as having had headaches, back pain, and stomach ache. Exploring differences in pain types, participants reported most often headache (44.6% at least once a week), back pain (20.0% at least once a week), and stomach ache (7.7% at least once a week).

The mean pain frequency sum score was 5.7 (SD = 3.1), reflecting that in average every participant reported two to three pain categories (headache, back pain, stomach ache, limb pain, joint pain, menstrual pain, teeth ache, and other pain) with an occurrence of 1–3 times a month or once a week. The mean pain intensity score was 1.2 (SD = 0.9). The mean impairment value was 2.6 (SD = 1.9).

Kolmogorov–Smirnov tests were performed to test these outcome variables for normal distribution. No significant results were obtained (mean pain frequency: test score 0.11, *p* = 0.11; mean pain intensity: test score 0.11, *p* = 0.15; mean impairment: test score 0.10, *p* = 0.20) allowing to use methods assuming normal distribution.

### Correlation analyses

Partial correlations between experimental pain measures and everyday pain experience can be found in Table 2. Overall, everyday pain and experimental pain experiences did correlate moderately: inverse correlation coefficients between pain threshold and everyday pain were observed with *r* = −0.25 (*p* = 0.09; pain frequency) to −0.35 (everyday pain intensity, *p* < 0.05). More pronounced and significant inverse correlation coefficients were found between mean pain tolerance and everyday pain frequency (*r* = −0.55, *p* < 0.001) as well as pain intensity (*r* = −0.34, *p* < 0.05).

**Table 2:** **Partial correlations between experimental pain measures, everyday pain experience and alexithymia subcomponents (*N* = 50)**.

Correlation Coefficient (*p*-value)	Pain threshold	Pair tolerance	Perceived pain intensity	Perceived pain unpleasantness	Everyday pain frequency	Everyday pain intensity	Everyday pain impairment	DDF	DIF EOT
Pain threshold	–								
Pain tolerance	0.50 (<0.001)	–							
Perceived pain intensity	−0.40 (<0.01)	−0.58 (<0.001)	–						
Perceived pain unpleasantness	−0.44 (<0.01)	−0.64 (<0.001)	0.49 (<0.001)	–					
Everyday pain frequency	−0.25 (0.09)	−0.55 (<0.001)	0.12 (0.40)	0.18 (0.23)	–				
Everyday pain intensity	−0.35 (<0.05)	−0.34 (<0.05)	0.32 (<0.05)	0.30 (<0.05)	0.39 (<0.01)	–			
Everyday pain impairment	0.16 (0.29)	−0.10 (0.52)	0.32 (<0.05)	0.28 (0.06)	−0.02 (0.87)	0.48 (<0.001)	–		
DDF	0.28 (0.07)	0.34 (<0.05)	−0.20 (0.18)	−0.24 (0.11)	0.10 (0.52)	−0.10 (−0.49)	−0.12 (0.51)	–	
DIF	0.10 (0.51)	0.20 (0.19)	−0.16 (0.29)	−0.21 (0.15)	0.03 (0.83)	−0.02 (0.91)	−0.18 (0.22)	0.59 (<0.001)	–
EOT	0.01 (0.93)	−0.11 (0.48)	−0.23 (0.13)	−0.15 (0.32)	−0.21 (0.16)	−0.24 (0.11)	−0.25 (0.09)	0.20 (0.19)	0.18 (0.24)

*Table shows partial correlation coefficients and *p*-values; warm threshold, age, EMI, and mood were controlled*.

**Figure 1:**
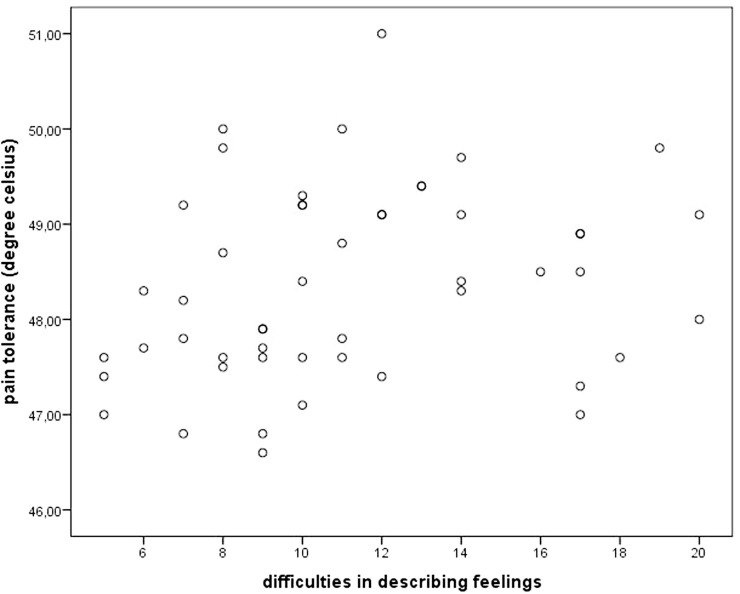
**Scatterplot depicting pain tolerance and alexithymia facet “difficulties in describing feelings”**.

**Figure 2:**
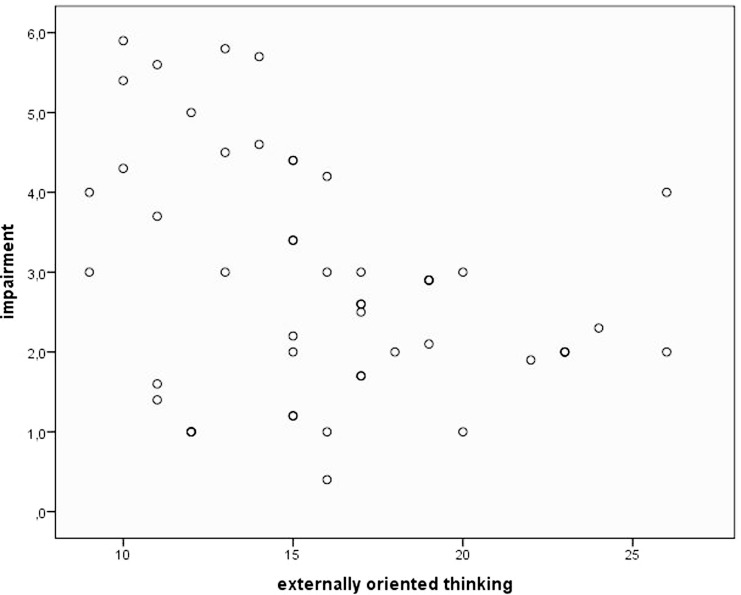
**Scatterplot depicting pain impairment and alexithymia facet “externally oriented thinking”**.

Concerning alexithymia, mean pain tolerance was positively related to the TAS subscales DDF (*r* = 0.34, *p* < 0.05). Figure 1 illustrates this relationship using a scatterplot. Everyday pain experience was only related to the EOT subscale, which was reflected in a trend toward an inverse correlation between EOT and pain impairment (*r* = −0.25, *p* = 0.09; see scatterplot Figure 2).

### Regression analyses

As experimental pain measures, everyday pain report and alexithymia were interconnected, we conducted five hierarchical regression analyses (forward stepping). Though only moderate correlation coefficients were found between alexithymia and pain measures, complex interrelations might mask important associations.

#### Experimental Pain Measures

Everyday pain measures (frequency, intensity, and impairment), thermal threshold (temperature sensitivity for heat), age, BMI, mood, and the three alexithymia subscales served as predictors. First, we wanted to clarify which variables explain significant proportion of variance of the criterion pain threshold. Warm threshold and mood explained a significant proportion of the criterion [*F*(2,47) = 12.25, *p* < 0.001, *R* = 0.59, *R*^2^ = 0.34]. Lower warm threshold (*T* = 3.94, β = 0.48, *p* < 0.001) and lower mood (*T* = 2.12, β = 0.26, *p* < 0.05) were associated with lower pain threshold.

Second, we used pain tolerance as criterion. Warm threshold (*T* = 2.05, β = 0.26, *p* < 0.05) and the alexithymia subscale DDF (*T* = 2.92, β = 0.38, *p* < 0.01) were significant predictors [*F*(2,47) = 7.03, *p* < 0.01, *R* = 0.48, *R*^2^ = 0.23]. High scores in DDF were associated with higher pain tolerance scores.

#### Everyday Pain

All regression analyses used experimental pain measures (threshold, tolerance, and thermal thresholds), age, BMI, mood, and the three alexithymia subscales as predictors. This is in accordance to former methodological approaches [see, e.g., Ref. (11)] and reflects found interrelation of these variables on pain measures [for age see, e.g., Ref. (34)].

First, everyday pain frequency pain was used as criterion; pain tolerance (*T* = −5.18, β = −0.59, *p* < 0.001) and the alexithymia subscale “difficulties in the identification of feelings” (*T* = 3.07, β = 0.35, *p* = < 0.01) and the BMI (*T* = –2.04, β = −0.23, *p* < 0.05) were significant predictors, all other predictors were not significant [*F*(3,46) = 12.69, *p* < 0.001, *R* = 0.66, *R*^2^ = 0.44]. High scores in DIF were associated with higher pain frequency in everyday life.

Then, everyday pain intensity served as criterion and was significantly explained by pain tolerance (*T* = −2.59, β = −0.35, *p* < 0.05) and EOT [*T* = −2.42, β = −0.32, *p* < 0.05; *F*(2,47) = 6.02, *p* < 0.01, *R* = 0.45, *R*^2^ = 0.20]. All other predictors were not significant. This means that high scores in EOT were associated with lower pain intensity in everyday life.

In a last regression, impairment due to everyday pain was used as criterion and was significantly explained by the alexithymia subscale EOT [*T* = −2.59, β = −0.35, *p* < 0.05; *F*(1,48) = 6.73, *p* < 0.05, *R* = 0.35, *R*^2^ = 0.12]. High scores in EOT were associated with low judged impairment due to everyday pain.

## Discussion

In accordance with our hypotheses, we found that the facets of alexithymia were differentially associated with experimental pain measures and reported everyday pain. While pain thresholds as a rather sensory-discriminative component were not affected by alexithymia, high scores on the emotional alexithymia subscale DDF were associated with *higher* pain tolerance reflecting *decreased* pain sensitivity. Importantly, only this affective facet of alexithymia interacted with the affective-motivational component of pain perception. In addition to the experimental assessment of pain, perception facets of alexithymia also modulated the experience of everyday pain with partly opposing effects. Regression analyses could demonstrate that the affective subscale DIF was *positively* related to everyday pain frequency, while high scores in the cognitive scale “externally oriented thinking” were associated with the *lower* impairment due to everyday pain. As to be expected, we found a significant positive relationship between pain intensity and pain unpleasantness in the experimental testing. This correlation coefficient was lower than in other studies, what could be related to the fact that we had repeated stimulus evaluations.

Our data extend former research by demonstrating that the alexithymia facets are differentially related to abnormalities in experimentally induced thermal pain processing and subjective everyday pain experience. Concerning experimentally induced pain we observed an positive correlation between one affective subscale of alexithymia and pain stimuli on the tolerance level which is in contradiction to former studies suggesting that high alexithymics have a low tolerance for electric (11) or visceral (35) pain stimulation. This correlation was mainly driven by those affective features of alexithymia that describe core problems of emotional awareness (23, 36). The fact that we did not obtain any effect of alexithymia facets on pain threshold is in accordance to data with eating disorder patients by de Zwaan and colleagues (37) and with fibromyalgia syndrome patients by Huber and co-authors (17). They reported that the degree of alexithymia did not influence thresholds to thermally and mechanically induced pain.

The study extends the knowledge about relationships between alexithymia facets and pain perception. It can be hypothesized that the different aspects of alexithymia interact with the capacity to regulate emotions and therefore with the perception of negative, painful stimuli. Externally oriented thinking was related to lower everyday pain suggesting that a greater tendency to look away from internal experience and seek for external sources might be helpful to some extent to deal with painful experiences. As different emotion regulation strategies can be distinguished [see, e.g., Ref. (38–40)], it is important to note that different subscales of alexithymia, including DDF, were found related to the strategy of expressive suppression [see also Ref. (41)]. DDF was the emotional subscale of alexithymia found to be associated with increased pain tolerance. Though suppression is associated with less positive and more negative emotional experience in general (39), Hampton and co-workers (42) could recently demonstrate that both reappraisal and suppression induction led to reductions in non-verbal and verbal indices of pain. In accordance to this result, also Lanzetta and co-authors (43) could show that emotion expressive suppression during anticipation of a painful stimulus decreased subsequent painfulness and skin conductance. It can be followed that for a while some alexithymic components also favor the coping with painful experience. Further research is necessary to elaborate what happens at higher level of alexithymia and starting at which level hampered affect regulation as observed in high alexithymics (13, 44, 45) might constitute a vulnerability factor for physical illness.

In this context, the idea that alexithymic facets are related to the perception of bodily signals is of great relevance. We suggest that when participants deal with emotionally modulated pain stimuli at tolerance level they use internal signals referring to changes in their bodily systems. Herbert and colleagues (19) could recently show that the accurate perception of bodily signals stemming from the cardiovascular system is indeed inversely related to emotional facets of alexithymia in a large sample of healthy individuals. Bodily signals and their perception (interoception) play an important role in many theories of emotions [e.g., Ref. (46–49)]. They are essential in the consolidation of somatic markers required for guiding individual behavior by signaling stimulus significance to the body as proposed in the somatic marker thesis by Damasio (46, 47). According to Damasio, degrees of conscious awareness are related to successive upgrades in the self representational maps (somatic markers) emerging from the feedback of bodily states. When emotional aspects of alexithymia are associated with difficulties in detecting bodily markers occurring during pain processing, regulation of pain will stronger depend on external cues. It can be hypothesized that alexithymic persons are, therefore, more prone to misinterpret their bodily sensations in, e.g., stressing situations which makes it more difficult for them to anchor their feelings correctly. This will lead to greater dependence on variables of the surrounding, possibly enhance the probability of errors like false alarms, i.e., that a not harmful stimulus is interpreted as potentially harmful and vice versa.

With respect to everyday pain, a complex pattern was observed: on the one hand, the alexithymia facet DIF was associated with a higher frequency of everyday pain. On the other hand, high scores in the cognitive component of alexithymia, as conceptualized in the EOT scale, are related to lower impairment. The fact that everyday pain frequency is positively related to the alexithymia – in detail the affective dimension DIF – is in accordance to former research (11, 16), suggesting that alexithymia is associated with over-reporting of physical symptoms, including pain, and a higher prevalence of chronic pain. De Gucht and colleagues (12) also reported the strongest association between somatic symptoms and the DIF subscale. This result fits nicely into studies highlighting that the three alexithymia facets are differentially linked to observed abnormalities in the processing of negative emotions (23–26). It can be argued that the emotional as well as the cognitive components of alexithymia might have differential effects on experimental pain measures and everyday pain. It is important to note that former research on somatoform patients highlighted alternations in experimental pain perception with several studies reporting a hypersensitivity to pain [e.g., Ref. (50)] while samples characterized by a high comorbidity with depression showed a hyposensitivity (51). It is, therefore, necessary to include depressive symptoms as a possible confounding variable in any future studies on alexithymia and pain.

Shibata and colleagues (16) argue that various theories linking alexithymia and physical illness have been conceptualized focusing at the physiological level (e.g., the hypothalamic-pituitary-adrenal axis, chronic sympathetic hyperarousal). Interestingly, some neuroimaging studies of alexithymia and chronic pain indicate not only hyperactivity in pain perception areas, such as the insular cortex, but also hypoactivity in pain-processing regulatory areas, such as the prefrontal cortex [e.g., Ref. (35)]. This observation fits into our interpretation that affective components of alexithymia are associated with a lack in emotional regulation ability that causes hypersensitivity to aversive bodily sensations. This is reflected by correlations between DIF and everyday pain probably associated with an individual’s ability to reduce or inhibit everyday pain. Nevertheless, the cognitive component of alexithymia as operationalized in the EOT scale was associated with rather low impairment by everyday pain and not related to experimentally induced pain, highlighting that it is important to assess alexithymia in more than one sum score.

We presented for the first time results showing that the facets of alexithymia are differentially associated with pain perception. These results may contribute to the understanding of the different aspects of alexithymia and many psychiatric and psychosomatic disorders known to be related to them. Further research with imaging data is necessary to investigate whether these observed differences are reflected in corresponding brain activation patterns. Potential limitations of the obtained results are that our findings focused on a small, healthy, and relatively young female population that exhibited scores below the critical cut-off scores for alexithymia. Future research is, therefore, necessary to demonstrate whether these preliminary results can be replicated with bigger samples, also with respect to male participants. Furthermore, only one kind of pain stimulation was used. Another restriction pertains to method used for pain induction and quantification. The tonic heat stimuli were relatively long and the single VAS rating after each stimulation phase did not allow detecting possible sensitization or habituation in the course of stimulation. In future studies, the application of additional methods, such as pressure or electric stimulation, may also be applied.

An important methodological problem refers to the fact that we asked the participants about their everyday pain experience in the last 2 months retrospectively. Such a subjective report procedure might be affected by a recall bias. It is well-known that emotions or the emotional state experienced at the time of memory retrieval can influence the information recalled, as, e.g., demonstrated for autobiographical retrieval (52) or for illness-related information in pain patients (53). Therefore, participants with current pain might report more painful experiences in the past weeks although we controlled for mood in all analyses carried out. Future studies might benefit from mobile assessment, such as implemented using mobile phones or smart watches when referring to everyday pain. A further methodological shortcoming refers to the fact that we did not assess depression in this sample. As depression and alexithymia are often found to be connected, depression might explain important mechanisms for the processing and evaluation of pain. Future studies should address this variable and its interaction with alexithymia, also in the subclinical range of both alexithymia and depressive symptoms.

As most of the former studies on alexithymia usually identified participants with high scores of alexithymia based on the TAS total score formed by the three facets of alexithymia, it can be concluded that a great part of the observed inconsistent results with regard to pain stimulation are due to this inadequate splitting procedure that is based on disregarding the multidimensionality of the construct alexithymia. Therefore, future research should focus on the facets of alexithymia in combination with different and well-defined experimental pain stimulation in order to access insight into the nature of alexithymia and its possible risk potential for psychiatric and somatic disorders.

## Conflict of Interest Statement

The authors declare that the research was conducted in the absence of any commercial or financial relationships that could be construed as a potential conflict of interest.
